# Onychomatricoma in a patient with skin of color

**DOI:** 10.1016/j.jdcr.2023.08.046

**Published:** 2023-09-15

**Authors:** Tejas P. Joshi, Carly Dunn, Yve T. Huttenbach, Soo Jung Kim

**Affiliations:** a School of Medicine, Baylor College of Medicine, Houston, Texas; b Department of Dermatology, Baylor College of Medicine, Houston, Texas; c Department of Pathology and Immunology, Baylor College of Medicine, Houston, Texas

**Keywords:** ethnic skin, nail matrix tumor, onychomatricoma, skin of color

*To the Editor*: We read with interest the report by Oak et al,^1^ who describe a case of onychomatricoma. Onychomatricoma is a rare, benign tumor of the nail matrix that is considered to almost exclusively affect patients with lighter skin types. Indeed, in a retrospective study of 30 patients with onychomatricoma, no cases were diagnosed in patients with dark brown skin—this is especially noteworthy since the study was conducted in Brazil and Miami, 2 regions with a relatively large proportion of patients of African descent.^2^ Moreover, nail conditions are poorly represented in skin of color (SoC) to begin with,^3^ further emphasizing a need to recognize and represent nail disorders in SoC. Here, we present the case of an African American patient with onychomatricoma.

A 56-year-old African American man presented for evaluation of his left second fingernail. Starting 13 years ago, he had progressive nail thickening with discoloration; he denied pain and pruritus. Physical examination of the left second fingernail disclosed a longitudinal thickened yellow nail plate with pronounced transverse curvature (Fig 1). Dermatoscopy of the nail plate revealed splinter hemorrhages, longitudinal white lines, and open spaces in a honeycombing configuration. A nail clipping was performed and sent for histopathology, which showed hyperkeratotic nail with round lacunae (Fig 2). Periodic acid–Schiff stain failed to demonstrate fungal elements. Based on the synthesis of clinical, dermatoscopic, and histologic findings, a diagnosis of onychomatricoma was made.

**Fig 1:**
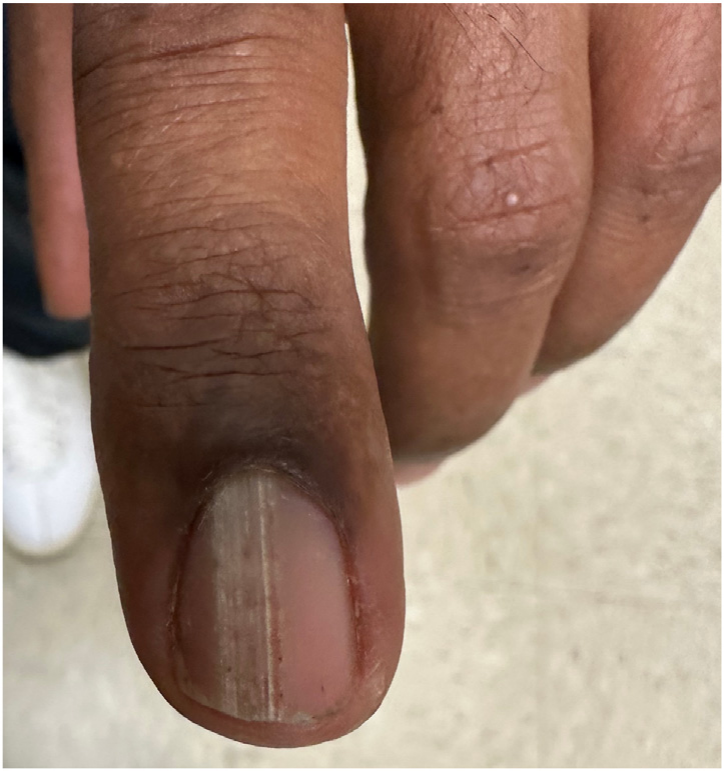
Left second fingernail shows thickened longitudinal ridge with xanthonychia and punctate hemorrhages.

**Fig 2:**
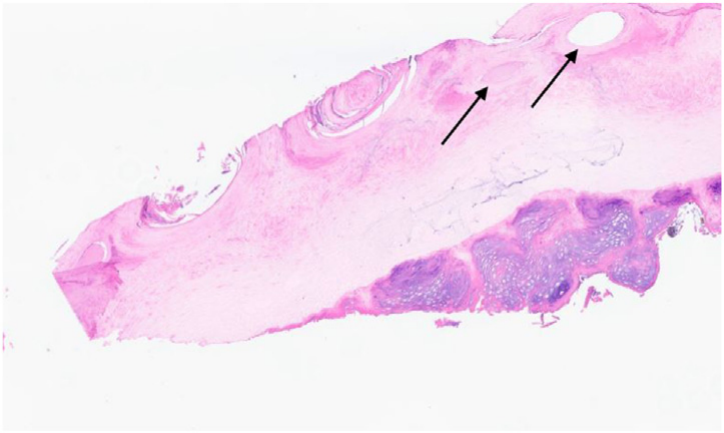
Histology of nail clipping displays hyperkeratotic nail with round lacunae, consistent with a diagnosis of onychomatricoma (hematoxylin and eosin, ×40 magnification).

Clinically, onychomatricoma presents with the tetrad of exaggerated transverse curvature, honeycombing, splinter hemorrhages, and xanthonychia. In addition to classic tetrad signs, longitudinal melanonychia, subungual hematoma, thickened proximal ungual fold, and dorsal pterygium may also be appreciated. It typically presents as a solitary lesion involving the fingernails. Onychomatricoma is often misdiagnosed as onychomycosis, and the differential diagnosis may include Bowen disease, fibrokeratoma, longitudinal melanonychia, osteochondroma, and subungual wart.^4^

A nail clipping is a minimally invasive technique to confirm the diagnosis of onychomatricoma. Histologic analysis of the nail clipping reveals lacunae filled with serous fluid. A periodic acid–Schiff stain can be used to exclude onychomycosis, and immunohistochemistry for cytokeratin may be performed as an adjunct to highlight the epithelial origin of the tumor.^4^ Imaging studies with ultrasonography and magnetic resonance imaging can supplement the diagnosis. Ultrasonography reveals a hypoechogenic area corresponding to the tumoral lesion and a hyperechogenic area corresponding to the digitiform projections. Magnetic resonance imaging demonstrates low uptake on the affected nail matrix with high uptake on fingerlike projections.^5^ Excisional biopsy with partial or complete nail avulsion is not necessary to establish a diagnosis, but may be performed therapeutically. As onychomatricoma is benign, treatment is elective.

Altogether, onychomatricoma is a rare, benign nail matrix tumor that presents with the clinical tetrad of exaggerated transverse curvature, honeycombing, splinter hemorrhages, and xanthonychia. Histologic examination of the nail clipping is sufficient to render a diagnosis of onychomatricoma. In addition to providing an image of onychomatricoma in SoC, our case report also emphasizes that onychomatricoma may occur in patients with darker skin types; thus, this diagnosis should not be discounted in SoC.

## Conflicts of interest

None disclosed.

## References

[1] Oak A.S.W., Elewski B.E., Pavlidakey P.G., Mayo T.T. (2020). Honeycomb-like cavities in a single fingernail plate. JAAD Case Rep.

[2] Di Chiacchio N., Tavares G.T., Tosti A. (2015). Onychomatricoma: epidemiological and clinical findings in a large series of 30 cases. Br J Dermatol.

[3] Falotico J.M., Lipner S.R. (2023). Lack of skin of color images of nail conditions in dermatology textbooks. Int J Dermatol.

[4] Perrin C., Baran R., Balaguer T. (2010). Onychomatricoma: new clinical and histological features. A review of 19 tumors. Am J Dermatopathol.

[5] Charfi O., Jaber K., Khammouma F. (2019). Magnetic resonance imaging in the diagnosis of onychomatricoma: a case report. Skin Appendage Disord.

